# Molecular Dynamics Simulation and Experimental Studies on the Thermomechanical Properties of Epoxy Resin with Different Anhydride Curing Agents

**DOI:** 10.3390/polym11060975

**Published:** 2019-06-03

**Authors:** Kexin Fu, Qing Xie, Fangcheng LÜ, Qijun Duan, Xinjie Wang, Quansheng Zhu, Zhengyong Huang

**Affiliations:** 1 State Key Laboratory of Alternate Electrical Power System with Renewable Energy Sources, North China Electric Power University, Baoding 071000, China; fkx_ncepu@foxmail.com (K.F.); 13582251628@126.com (F.L.); duan_ncepu@163.com (Q.D.); 2 Department of English, North China Electric Power University, Baoding 071000, China; July2010@126.com; 3 State Grid Henan Electric Power Company, Zhengzhou 450052, China; zhuquansheng@ha.sgcc.com.cn; 4 State Key Laboratory of Power Transmission Equipment & System Security and New Technology, Chongqing University, Chongqing 400044, China; huangzhengyong@cqu.edu.cn

**Keywords:** epoxy resin, anhydride curing agents, molecular dynamics simulation, thermomechanical properties, structure-property relationship

## Abstract

An investigation of the relationship between the microstructure parameters and thermomechanical properties of epoxy resin can provide a scientific basis for the optimization of epoxy systems. In this paper, the thermomechanical properties of diglycidyl ether of bisphenol A (DGEBA)/methyl tetrahydrophthalic anhydride (MTHPA) and DGEBA/nadic anhydride (NA) were calculated and tested by the method of molecular dynamics (MD) simulation combined with experimental verification. The effects of anhydride curing agents on the thermomechanical properties of epoxy resin were investigated. The results of the simulation and experiment showed that the thermomechanical parameters (glass transition temperature (*T*_g_) and Young’s modulus) of the DGEBA/NA system were higher than those of the DGEBA/MTHPA system. The simulation results had a good agreement with the experimental data, which verified the accuracy of the crosslinking model of epoxy resin cured with anhydride curing agents. The microstructure parameters of the anhydride-epoxy system were analyzed by MD simulation, including bond-length distribution, synergy rotational energy barrier, cohesive energy density (CED) and fraction free volume (FFV). The results indicated that the bond-length distribution of the MTHPA and NA was the same except for C–C bonds. Compared with the DGEBA/MTHPA system, the DGEBA/NA system had a higher synergy rotational energy barrier and CED, and lower FFV. It can be seen that the slight change of curing agent structure has a significant effect on the synergy rotational energy barrier, CED and FFV, thus affecting the *T*_g_ and modulus of the system.

## 1. Introduction

Diglycidyl ether of bisphenol A (DGEBA) reacts with an anhydride curing agent to form a three-dimensional crosslinked network structure, which has the advantages of excellent insulation performance, superior mechanical properties, easy processing and low cost. It is widely used in high voltage insulation systems [1,2,3,4,5]. Composites prepared by filling modification are usually used to improve the thermomechanical properties of epoxy resins [6,7,8,9,10]. However, filling of a large number of fillers will lead to decreased insulation properties [11]. If epoxy resins with outstanding thermomechanical performance can be prepared, the loading of filler will reduce dramatically, which lays the foundation to obtain epoxy resin composites with excellent comprehensive properties. On the premise of ensuring high insulation performance of epoxy resin, how to improve thermomechanical properties is a great challenge and key technology in the field of electrical materials.

Epoxy resin performance depends on the molecular structure of the crosslinked network [12,13,14]. The molecular structure of the crosslinked network can be achieved by changing the crosslinking density [15,16] and molecular structure of curing agent [13,14,17,18,19]. Epoxy polymers synthesized with different curing agents have different thermal and mechanical properties [14,17,19,20]. There are many kinds of epoxy resins and curing agents, but screening high performance epoxy resins by experiments is time-consuming, expensive and has low efficiency, plus it is difficult to analyze the relationships between molecular structure and macroscopic properties of epoxy resin networks [3,13,21]. Molecular dynamics (MD) simulations combined with experimental research methods have the advantages of shortening the development cycle and saving costs in predicting the properties of thermosetting epoxy resin materials. Meanwhile, a new feasible method has been provided to explore the relationship between the structure and properties of epoxy resins [14,21]. 

In recent years, MD simulation technology has received much attention from researchers. Researchers have used MD simulation technology to conduct much research on the performance prediction and theoretical exploration of thermosetting epoxy resins. Research on the thermomechanical properties of epoxy resins is mainly focused on the crosslinked network structures formed by epoxy resins cured with amine curing agents [1,2,13,14,17,18,20,22,23,24,25]. Zhang et al. [18] calculated the theoretical modulus of the DETDA/TDE85 and MPD/TDE85 epoxy matrix through MD simulation. The flexibility and fluidity of molecular chains and the fraction free volume (FFV) of epoxy resins were analyzed in detail. It was revealed that not only was the stiffness of molecules an influential factor on the modulus of crosslinked epoxy resin systems, but also intermolecular forces and molecular chain packing structures. Zhang et al. [22] investigated the glass transition temperature (*T*_g_) and modulus of DDM/DGEBA and DDS/DGEBA epoxy resin with the help of MD simulation combined with experimental verification. The results showed that the flexibility of curing-agent molecules directly affects the properties of materials. The thermal and mechanical properties of DGEBA/TETA and DGEBA/DETDA epoxy resin systems have also been studied by F. Jeyranpour et al. [1]. Yang et al. [13] conducted MD simulation to investigate the relationship between microstructure and macro-properties of DDM/TDE85 and DDS/TDE85. By analyzing conformational graphs and cohesive energy density (CED), the effects of a slight modification of amine structure on *T*_g_ were revealed. However, most simulation studies rarely involve the anhydride-epoxy systems widely used in electrical insulating materials; specifically, there are no reports on the effects of different anhydride curing agents on the thermomechanical properties of their epoxy-cured products, or that analyze the relationship between microstructures and properties.

In this paper, we calculated the mechanical and thermal properties of DGEBA/methyl tetrahydrophthalic anhydride (MTHPA) and DGEBA/ nadic anhydride (NA) epoxy resins with a crosslink density of 89% using MD simulation. The samples of DGEBA/MTHPA and DGEBA/NA epoxy resins were prepared and their thermomechanical performances were measured. We investigated the effects of anhydride curing agents with different molecular structures on the thermomechanical properties of epoxy resins. The relationship between molecular structure and properties of epoxy resin networks was studied by analyzing bond-length distribution, synergy rotational energy barrier, CED and FFV.

## 2. Methodology

### 2.1. Simulation

The models of DGEBA/MTHPA and DGEBA/NA crosslinked networks were constructed to investigate the effect of different anhydride curing agents on the thermomechanical performances of epoxy resins, and to analyze the relationship between molecular structure and properties. The models in this paper were built using Materials Studio 7.0 developed by Accelrys (San Diego, CA, USA).

#### 2.1.1. Crosslinking Mechanism of Epoxy Resin and Anhydride

**Figure 1:**
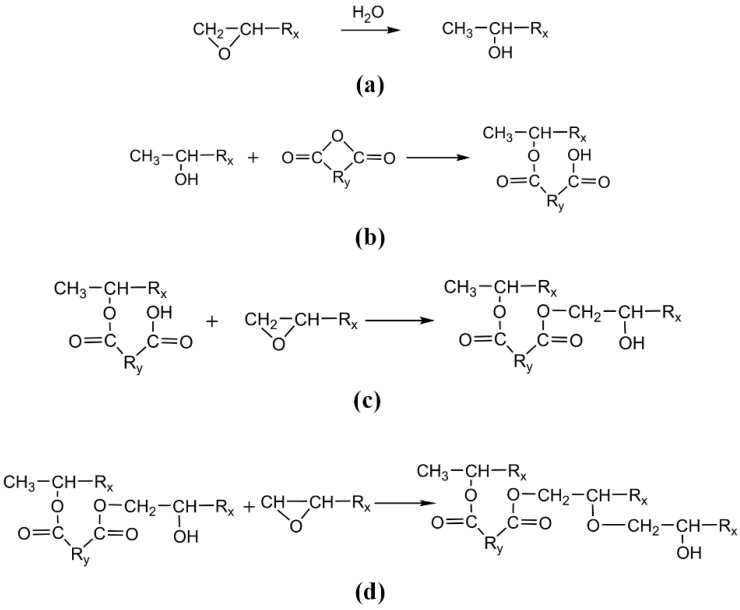
Curing reaction mechanism. (**a**) Micro-water opens the epoxy group to form a hydroxyl group; (**b**) hydroxyl and anhydride group produces monoester; (**c**) carboxyl and epoxy group forms diesters; (**d**) hydroxyl and epoxy groups etherify.

The commonly accepted epoxy-anhydride reaction mechanism suggests that the reaction is an alternate ring-opening polymerization of anhydrides and epoxies [26]. Researches have shown that at high temperatures, in the presence of catalysts or with an excess of epoxy, homopolymerization of epoxy groups also occurs [19,27,28]. In this paper, the curing temperature was below 473.15 K and the DGEBA was not excessive; therefore, the curing reaction mechanism of epoxy-anhydride did not include homopolymerization. The curing reaction mechanism of epoxy-anhydride is shown in Figure 1. Without an accelerating agent, the main reactions between anhydride and epoxy resin were as follows: (1) micro-water opened the epoxy group to form a hydroxyl group, (2) hydroxyl and anhydride group produced monoester, (3) carboxyl and epoxy group formed diesters and (4) etherification of hydroxyl and epoxy groups was conducted [5,26]. Though the above main reactions, the epoxy resin 3D crosslinked network was finally formed.

#### 2.1.2. Establishment of Models

**Figure 2:**
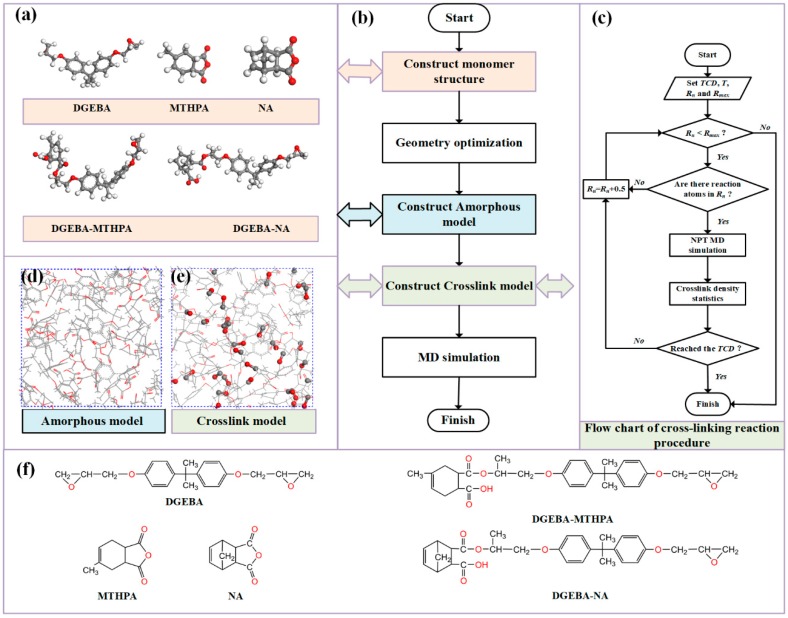
Establishment of molecular models. (**a**) Molecular structure of DGEBA, MTHPA, NA, DGEBA-MTHPA and DGEBA-NA; (**b**) flow diagram of construction; (**c**) flow chart of crosslinking reaction program; (**d**) amorphous model, (**e**) crosslink model; (**f**) molecular formulas of DGEBA, MTHPA, NA, DGEBA-MTHPA and DGEBA-NA.

The modeling process is shown in Figure 2, and the details are as follows:

(1) The molecular models of monomer (DGEBA, MTHPA, NA) and primary crosslinking structure (DGEBA-MTHPA and DGEBA-NA) were constructed, respectively. Their molecular structures and molecular formulas are shown in Figure 2a,f. Considering the actual average degree of polymerization (PD) of DGEBA in the experimental study, PD was between 0.1 and 0.2 [5]. Therefore, when building the DGEBA monomer model, the PD was set to zero. 

(2) The 3D amorphous models of DGEBA/MTHPA and DGEBA/NA were constructed using the Amorphous Cell Tools, as shown in Figure 2d. The research results of [29] indicate the cell size had no large effect on the calculated properties, while [3,30] investigated the thermomechanical properties of epoxy resin using the small system (the amorphous cells containing 4, 8, 12 and 16 DGEBA). Meanwhile, to verify the accuracy of the model, the DGEBA/MTHPA system containing 40 DGEBA, 90 MTHPA and 10 DGEBA-MTHPA was constructed [31]. The *T*_g_ of this system was 400.46 K at a crosslinking density of 89%. The result was consistent with the result of the small system. Therefore, to reduce the computational requirements, the epoxy resin system amorphous models were constructed using the Calculation option in Amorphous Cell Tools. The initial parameters of each epoxy resin model can be seen in Table 1. Ten amorphous models were constructed for each system. The molar ratio of the epoxy group to the anhydride group was 1:1 for each model with an initial density of 0.6 g/cm^3^.

(3) After geometric structure optimization, the amorphous models with lowest energy were selected for further simulation. In order to eliminate the stress generated in the process of modeling and make the model density conform to the actual situation, MD simulation was subsequently conducted in constant volume and temperature (NVT) ensemble for 100 ps at 300 K. Afterwards, the model was equilibrated in constant pressure and temperature (NPT) ensemble for 200 ps at 1 atm. In MD simulation, the time step was 1 fs, the force field COMPASS was selected and the Charges, Electrostatic and van der Waals were set as Forcefield assigned, Ewald and Atom based, respectively. The Andersen and Berendsen were chosen to control temperature and pressure, respectively.

(4) The script of the automatic crosslinking reaction program for epoxy resin/anhydride curing agent was written according to the mechanism of the acid anhydride curing reaction in Section 2.1.1, as shown in Figure 1. To simplify the process, the following assumptions were made [19,31]: (i) The step (1) in Section 2.1.1 was omitted, and the carboxyl in primary crosslinking structure reacted with the epoxy group as the initial reaction site. (ii) The reactivity of each reaction group was the same. (iii) The reactions were diffusion-controlled. (iv) The reactions were synchronized. The flow chart of cross-linking reaction program [31] is shown in Figure 2c, where *TCD*, *T*, *R*_n_ and *R*_max_ are target crosslink density, temperature, truncation radius and maximum truncation radius, respectively. *T* was set at 300 K, and *R*_n_ and *R*_max_ were set at 3.5 and 7.5 Å, respectively. The crosslinking structure model of epoxy resin can be obtained by setting parameters, as shown in Figure 2e.

#### 2.1.3. Simulation Details

Typically, conversion degrees in well-controlled curing processes are in the 80–95% range [14]. Meanwhile, the different crosslinking density models were calculated before determining the investigation model. The results showed thermomechanical performance was excellent when the crosslinking density was 89%. Therefore, the crosslinking density of 89% was used for analysis. 

After optimizing the structure of the crosslinking model with the crosslink density of 89%, MD simulation was conducted successively in NVT and NPT ensembles at 600 K, and the simulation parameters were consistent with step (3) in Section 2.1.2. Finally, the crosslinking model of 600 K was annealed and the crosslinking model of epoxy was achieved with different temperatures. Finally, the crosslinking model of 600 K was annealed to 300 K at a cooling rate of 50 K/100 ps [1,32,33], and the crosslinking structure parameters of epoxy resin at different temperatures were obtained.

The last 30 frames selected from the annealed files of epoxy resin systems with crosslink density of 89% were calculated five times, and the mean value was taken as the theoretical modulus. The average values of specific volumes of the last 30 frames selected from the annealed files at different temperatures were calculated, and the *T*_g_ was achieved using a specific volume–temperature fitting curve.

### 2.2. Experimental

In order to verify the accuracy of the crosslinking model of the acid anhydride-epoxy system, samples of DGEBA/MTHPA and DGEBA/NA epoxy resin were prepared. Shanghai Resin Factory, Shanghai, China, supplied the epoxy resin DGEBA (epoxy value, 0.5–0.54) and the curing agent MTHPA. Aladdin Reagent Co., Ltd. (Shanghai, China) supplied the curing agent NA. The accelerator 2,4,6-tri (dimethyl amino methyl) phenol (DMP-30) (amine value 590–650 mg/g) was supplied by Shanghai Resin Factory, Shanghai, China. The specific experimental process was as follows. Using the DGEBA/MTHPA system as an example, the mass ratio of DGEBA, MTHPA and MPD30 was 100:80:1, respectively. The curing agent MTHPA was mixed with th epoxy resin DGEBA in the reaction kettle by using mechanical stirring at 400 rpm under 343.15 K for 1 h. Subsequently, the accelerator MPD30 was added to the reaction kettle and then the mixture was stirred at 400 rpm under 333.15 K for 30 min. After that, the above mixtures were degassed at 313.15 K for 30 min under a vacuum. Finally, the resin systems were poured into a steel mold and cured via 413.15 K for 1 h. After mold unloading, the samples were cured at 393.15 K for 10 h. After cooling down to room temperature in the oven, the samples of different epoxy systems were obtained.

The *T*_g_ was measured via Q10 differential scanning calorimetry (DSC) by TA Instruments (New Castle, DE, USA). The range of temperature rise was from room temperature to 453.15 K, and the rate of temperature rise was 10 K/min. According to GB/T2567-2008, the tensile performance of resin casts was obtained by using a universal testing machine (HZ-1003, Dongguan, Guangdong, China) with a testing speed of 2 mm/min to investigate the Young’s modulus of different samples. To ensure the accuracy of the data, 10 specimens were tested for every case and the average values were taken.

## 3. Results and Discussions

### 3.1. Bond-Length Distribution Analysis

**Figure 3:**
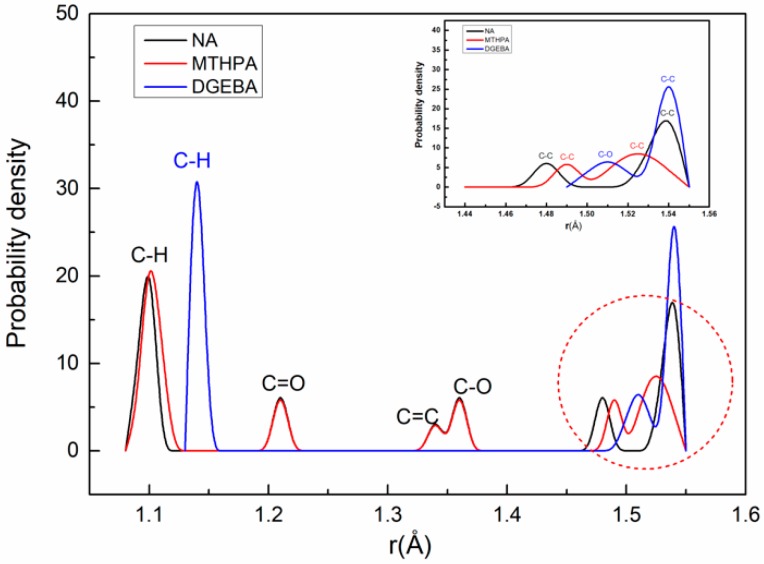
The bond-length distribution of DGEBA, MTHPA and NA monomers.

In this section, the bond-length distributions of the monomer (DGEBA, MTHPA and NA) and the primary crosslinking structure (DGEBA-MTHPA and DGEBA-NA) are calculated and analyzed. Figure 3 shows the bond-length distribution of DGEBA, MTHPA and NA monomers. As can be seen from Figure 3, the bond-length distributions of C–H, C=O, C=C and C–O bonds of MTHPA and NA monomers are basically the same, and the bond-length distributions of C–C bonds are quite different.

**Figure 4:**
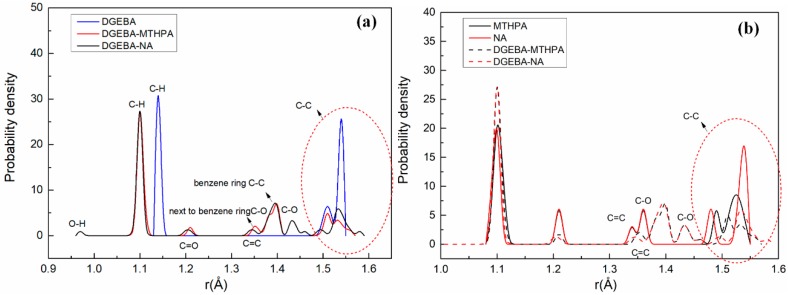
Comparison of bond-length distributions. (**a**) The primary crosslinking structure and the epoxy resin DGEBA; (**b**) the primary crosslinking structure and the curing agents MTHPA and NA.

**Figure 5:**
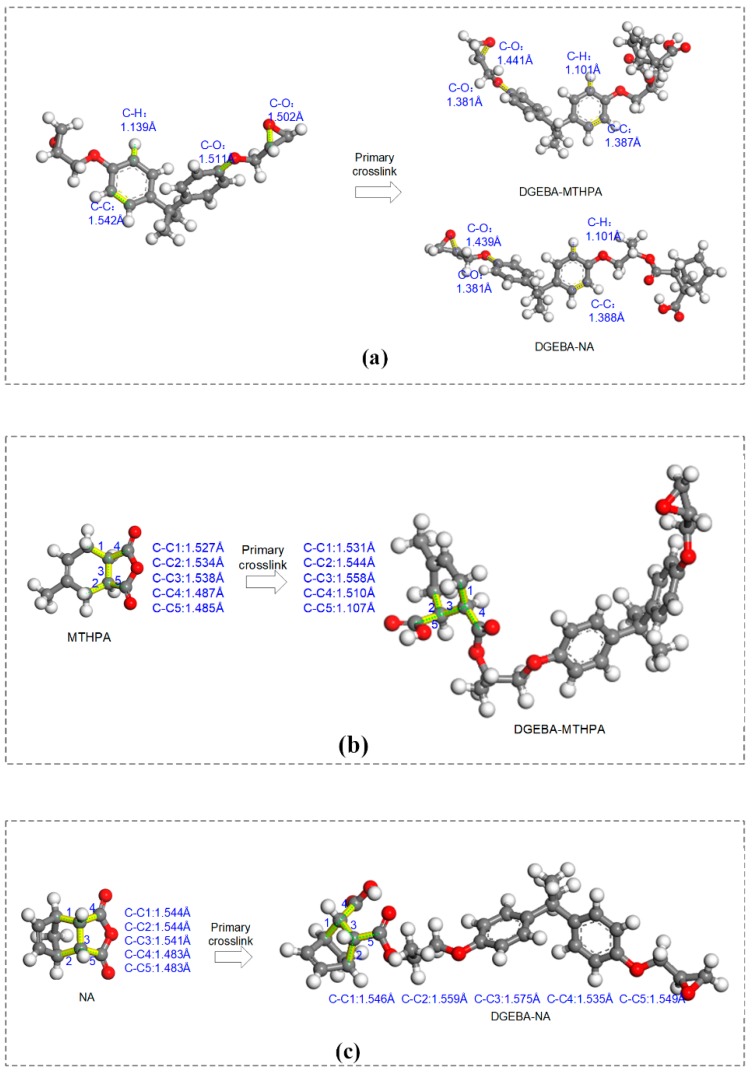
Specific bond length changes between the monomers and primary crosslinking structures. (**a**) The primary crosslinking structure and the epoxy resin DGEBA; (**b**) the primary crosslinking structure and the curing agent MTHPA; (**c**) the primary crosslinking structure and the curing agent NA.

A comparison of bond-length distribution between the primary crosslinking structure (DGEBA-MTHPA and DGEBA-NA) and the monomer (DGEBA, MTHPA and NA) is shown in Figure 4. As can be seen in Figure 4a, compared with DGEBA, the bond lengths of C–H bonds of the DGEBA-MTHPA and DGEBA-NA primary crosslinking structures and C– bonds on benzene rings shifted the same distance to the left. Figure 4b shows that the C=C bonds and C–O bonds of the primary structure (DGEBA-MTHPA and DGEBA-NA) moved to the right compared with curing-agent monomers. Moreover, the change of C–C bonds is complicated. The specific bond length changes between the monomers and primary crosslinking structures are shown in Figure 5. The change values of the specific bonds length can be seen in Figure 5. The analysis indicates that the original structure of the monomers will change after forming the epoxy crosslinked structure.

### 3.2. Conformation and Cohesive Energy Analysis

**Figure 6:**
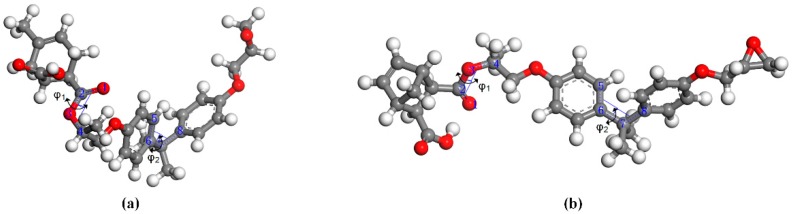
The torsional rotation of the specific bond. (**a**) DGEBA-MTHPA; (**b**) DGEBA-NA.

**Figure 7:**
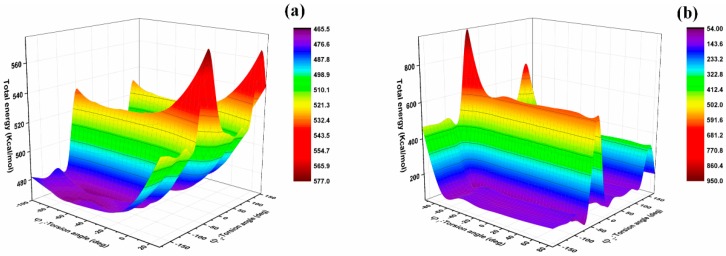
The potential energy graph of the bonds φ1 and φ2. (**a**) DGEBA-MTHPA; (**b**) DGEBA-NA.

It is well known that the flexibility of molecular segments can exert an important influence on the thermomechanical performance of epoxy resin. Flexible chains will result in low *T*_g_ and low modulus [13,18]. The synergy rotational energy barrier can be inverted from the energy range associated with a particular build-up torsion; the larger the synergy rotational energy barrier, the worse the flexibility of the chain [13,22]. The analysis of bond-length distribution shows that the original molecular structure of the monomer changes after the formation of the crosslinking structure. Therefore, the analysis of the synergy rotational energy barrier of the specific bond of the primary crosslinking structure can better reflect the flexibility of the chain segment. Analysis of conformation behavior of molecular structure can provide useful information on the local structure and chain flexibility in the crosslinking network of the epoxy resins [18]. For the conformational grid search, the energies associated with torsional rotations of specific bonds can be obtained. The rotational energy barrier of a single bond cannot indicate the flexibility of molecular chains, and therefore cooperative motions of bonds need to be paid attention to as the synergy rotational energy barrier of the specific bond is calculated. The torsional rotation of the specific bonds *φ*_1_ and *φ*_2_ is illustrated in Figure 6. The potential energy graph of bonds *φ*_1_ and *φ*_2_ in the DGEBA-MTHPA and DGEBA-NA primary crosslinking structure are shown in Figure 7. The specific synergy rotational energy barrier of the DGEBA-MTHPA primary crosslinking structure was 107.62 kcal/mol, and that of the DGEBA-NA primary crosslinking structure was 832.02 kcal/mol, which was higher than that of the DGEBA-MTHPA primary crosslinking structure. 

The CED of polymers can be used to roughly predict thermal and mechanical properties of materials; a smaller CED generally implies a lower *T*_g_ and theoretical modulus [13,18]. The cohesive energy (*E*_coh_) of a system of molecules is the average energy required to separate all molecules to an infinite distance from each other. *E*_coh_ is calculated by the follow equation [13]:

(1) $$E_{\mathrm{coh}}=\langle E_{\mathrm{inter}}\rangle =\langle E_{\mathrm{total}}\rangle -\langle E_{\mathrm{intra}}\rangle$$

where *E*_inter_ is the total energy between all molecules, *E*_total_ is the total energy of a system and *E*_intra_ is the intramolecular energy. The brackets <···> represent an average over a constant temperature and constant pressure (NPT) ensemble or constant temperature and constant volume (NVT) ensemble.

The CED is simply the cohesive energy per unit of volume [18]:

(2) $$\mathrm{CED}=\frac{E_{\mathrm{coh}}}{V}$$

The CED of the DGEBA/MTHPA and DGEBA/NA systems is displayed in Table 2. It was found that the CED of DGEBA/MTHPA was about 464.12 J/cm^3^, which was lower than that of DGEBA/NA, specifically, 475.50 J/cm^3^. It was indicated that intermolecular force of DGEBA/MTHPA was smaller than that of DGEBA/NA. 

By the analysis above, it is believed that the molecular chain flexibility of the DGEBA/NA epoxy resin system was worse and its CED was higher. It can be predicted that the *T*_g_ and modulus of DGEBA/MTHPA was lower than that of the DGEBA/NA. This is consistent with the simulation and experimental results (see Table 3 and Table 4)

### 3.3. Free Volume Analysis

The influence of curing-agent structure on FFV is very prominent [18]. The investigation of free volume at a molecular scale is helpful to understand the accumulation and spatial state of polymer molecules. According to free volume theory [34], the volume (*V_T_*) of a liquid or solid material consists of two parts, the volume occupied by molecules (i.e., the actual occupancy volume (*V_0_*)) and the unoccupied volume (i.e., the free volume (*V*_f_)).

(3) $$V_{T}=V_{0}+V_{f}$$

FFV is introduced to represent the relative size of the free volume of different polymers, and can be calculated by the following equation [35]:

(4) $$\mathrm{FFV}=\frac{V_{f}}{V_{0}+V_{f}}\times 100\%$$

**Figure 8:**
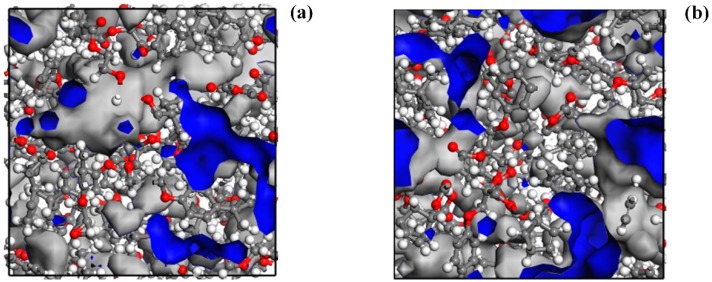
Free volume of (**a**) DGEBA-MTHPA; (**b**) DGEBA-NA.

Free volume is an important factor affecting the thermomechanical properties of epoxy resins. The reduction of free volume in a glass state can inhibit the movement of molecular chains and lead to a higher modulus and *T*_g_. The free volume of epoxy resin depends on the packing ability of molecular chains and the geometric constraints imposed by the crosslinked network [36]. In this paper, the free volume was acquired by calculating the Connolly surface through the Atom Volume & Surface modules. Figure 8 shows the free volume of DGEBA-MTHPA and DGEBA-NA with a crosslinking degree of 89% at 300 K.

The FFV of the epoxy system with the crosslink density of 89% at 300 K is displayed in Table 3. It was found that the FFV of the DGEBA-NA system was higher than that of the DGEBA-MTHPA system, and the structure of the DGEBA-NA system was more compact, caused by the steric hindrance effect of methyl in MTHPA. It can be predicted that the *T*_g_ and modulus of DGEBA/NA was higher than that of DGEBA/MTHPA. This is consistent with the simulation and experimental results (see Table 3 and Table 4)

### 3.4. Thermal Properties Analysis

**Figure 9:**
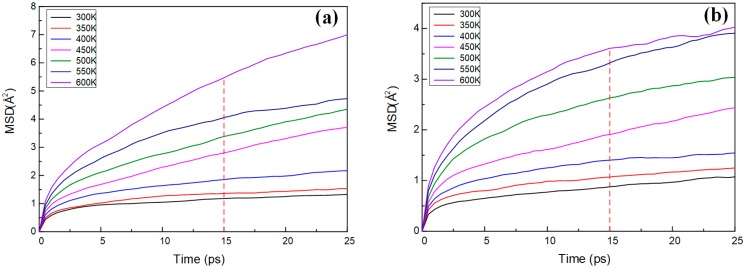
The MSD-time curves of epoxy resins. (**a**) DGEBA-MTHPA; (**b**) DGEBA-NA.

**Figure 10:**
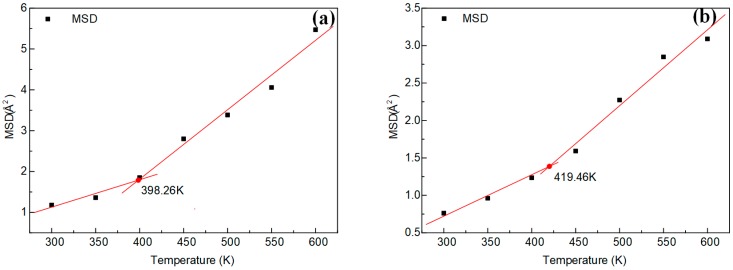
The MSD (15 ps)-temperature fitting curves of epoxy resins. (**a**) DGEBA-MTHPA; (**b**) DGEBA-NA.

*T*_g_ is an important parameter of polymer materials. At *T*_g_, the behavior of a polymer shifts from a glassy and brittle state to a rubbery state [1], accompanied by changes in physical properties such as specific volume and thermal, mechanical and electrical properties. Therefore, the *T*_g_ can be obtained either by linear fitting of the specific volume and temperature, or by the mean squared displacement (MSD) curve fitting [30,37]. The MSD for a system of N atoms can be described by the following equation [22,38]:

(5) $$\mathrm{MSD}=\frac{1}{3N}\sum_{i=0}^{N-1}\langle {\left|\left(\vec{R_{i}}\right)\left(t\right)-\left(\vec{R_{i}}\right)\left(0\right)\right|}^{2}\rangle$$

where $\left(\vec{R_{i}}\right)\left(t\right)$ and $\left(\vec{R_{i}}\right)\left(0\right)$ represent the displacement vectors of any atom *i* in the system at time *t* and initial time, respectively. When a polymer undergoes a glassy-to-rubbery phase transition, the torsional and rotational motions of the molecules combined with the local motion can cause chain disentanglement, leading to a sudden increase in diffusivity and, consequently, a sudden jump in the MSD-time curves obtained at a range of temperatures [39]. MSD-time curves are shown in Figure 9. The glass transition region can be roughly estimated by searching for an unusually large gap between MSD-time curves [37,39]. The inflection point was obtained by fitting the MSD value-temperature curve at 5, 10, 15, 20 and 25 ps, respectively. The calculation value at MSD-15 ps is consistent with the *T*_g_ obtained by the specific volume-temperature fitting curve. Therefore, the inflection point obtained by fitting the MSD value at 15 ps with the temperature can be regarded as the approximate value of *T*_g_. The *T*_g_ achieved by the MSD (15 ps)-temperature fitting is shown in Figure 10.

**Figure 11:**
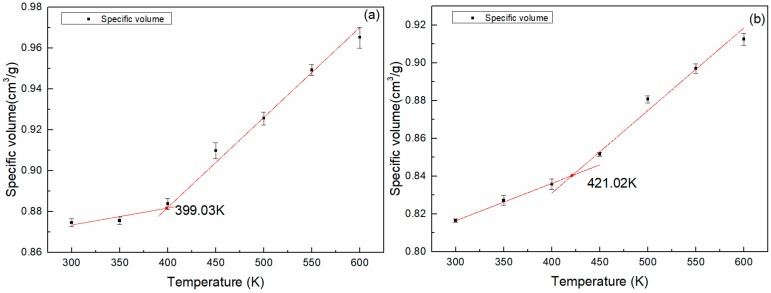
The specific volume-temperature fitting curves of epoxy resins. (**a**) DGEBA-MTHPA; (**b**) DGEBA-NA.

Figure 11 shows the *T*_g_ fitting curves of specific volume-temperature. Figure 10 and Figure 11 show that the *T*_g_ obtained by the two fitting methods were almost the same. The *T*_g_ of the DGEBA/NA system was 19.83 K higher than that of the DGEBA/MTHPA system when the specific volume-temperature fitting was used. The *T*_g_ of the DGEBA/NA system achieved by the MSD (15 ps)-temperature fitting was 21.22 K higher than that of DGEBA/MTHPA system. The results of the two methods were nearly identical.

It can be seen from Table 4 that the simulation and experimental results of the *T*_g_ of the DGEBA/NA system were higher than those of the DGEBA/MTHPA system, which is consistent with the *T*_g_ results predicted by the synergy rotational energy barrier, CED and FFV analyses.

### 3.5. Mechanical Properties Analysis

In this paper, the mechanical properties of epoxy resins were calculated by a static constant strain method [2,17]. The process can be described as follows. For systems that have reached mechanical equilibrium, a slight strain is applied to the system, which causes uniaxial tension and compression deformation along the *x*-, *y*- and *z*-axis of the three dimensional epoxy system, and generates shear deformation in the *xy*-, *xz*- and *yz*- planes. The stress–strain relationship within the realm of linear elastic obeys Hooke’s law:

(6) $$\sigma_{i}=C_{ij}\varepsilon_{j}$$

where *σ_i_* and *ε_j_* are stress and strain vectors, respectively, and *C_ij_* is the 6-dimensional stiffness matrix. In the simulation, the epoxy models can be assumed isotropic. Therefore, *C_ij_* can be simplified as follow [2]:

(7) $$C_{ij}=\left[\begin{array}{cccccc} \lambda +2\mu & \lambda & \lambda & 0 & 0 & 0\\ \lambda & \lambda +2\mu & \lambda & 0 & 0 & 0\\ \lambda & \lambda & \lambda +2\mu & 0 & 0 & 0\\ 0 & 0 & 0 & \mu & 0 & 0\\ 0 & 0 & 0 & 0 & \mu & 0\\ 0 & 0 & 0 & 0 & 0 & \mu \end{array}\right]$$

where *λ* and *μ* are the elastic constant, and can be obtained from the stiffness matrix.

(8) $$\left\{ \begin{array}{l} \lambda =\frac{1}{6}\left(C_{12}+C_{13}+C_{21}+C_{23}+C_{31}+C_{32}\right)\\ \mu =\frac{1}{3}\left(C_{44}+C_{55}+C_{66}\right) \end{array}\right.$$

The parameters such as bulk modulus *K*, Young’s modulus *E*, shear modulus *G* and Poisson’s ratio *ν* of the epoxy resins can be obtained from *λ* and *μ* [17].

(9) $$\left\{ \begin{array}{l} K=\lambda +\frac{2}{3}\mu \\ E=\mu \frac{3\lambda +2\mu }{\lambda +\mu }\\ G=\mu \\ \upsilon =\frac{\lambda }{2\left(\lambda +\mu \right)} \end{array}\right.$$

The mechanical properties of the DGEBA/MTHPA and DGEBA/NA systems are calculated as shown in Table 5. It can be seen from Table 5 that the theoretical modulus of the DGEBA/NA system is higher than that of the DGEBA/MTHPA system.

**Figure 12:**
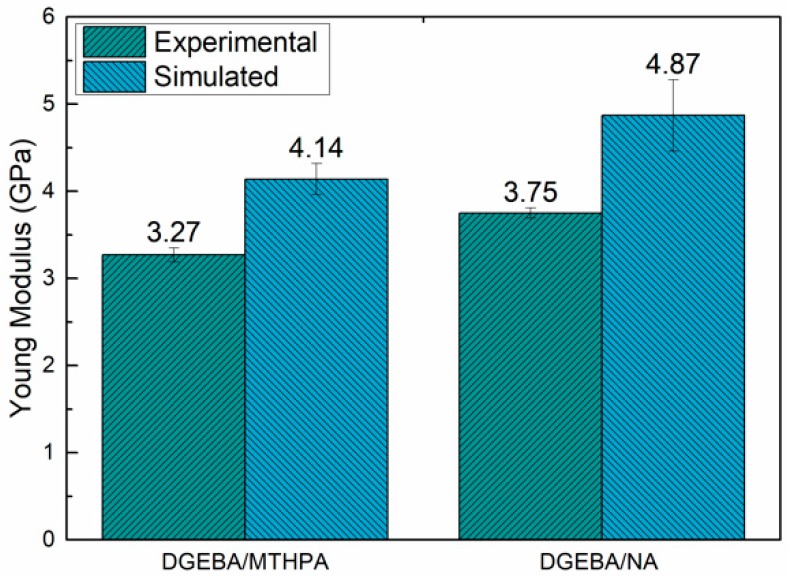
The simulation and experimental results of Young’s modulus.

Then the simulation results of the DGEBA/MTHPA and DGEBA/NA systems are compared with the experimental results. As can be seen from Figure 12, the modulus of the DGEBA/NA systems obtained by simulation and experimental tests was higher than that of DGEBA/MTHPA systems. This is consistent with the predicted results of the theoretical modulus of the synergy rotational energy barrier, cohesive energy density and free volume analyses. Nevertheless, the simulation values were higher than the values of experimental measurement, which may have been caused by the simulation model’s ideal crosslinking structure, composition [18] and model scale [40], in contrast with the inevitable structural defects and other factors existing in the actual system.

## 4. Conclusions

In this paper, the effects of anhydride curing agents MTHPA and NA on the thermomechanical properties of epoxy resin were studied by molecular dynamics simulations and experimental verification. The relationship between the molecular structure of epoxy resin and its thermomechanical properties was analyzed. The results are as follows: 

(1) The bond-length distributions of MTHPA and NA curing-agent monomers were quite the same, except for the C-C bond. Compared with DGEBA, MTHPA and NA monomers, the bond-length distribution of corresponding monomers in the primary crosslinking structure of MTHPA and NA will change after the formation of primary crosslinking structures. The analysis shows that the slight change of molecular structure of the anhydride curing agent has a great influence on the properties of epoxy crosslinked networks.

(2) Compared with the DGEBA/MTHPA system, MD simulation results showed that the synergy rotational energy barrier and CED of the DGEBA/NA system were larger, the FFV of the DGEBA/NA system was smaller and the calculated values of the *T*_g_ and theoretical modulus of the DGEBA/NA system were higher. In other words, the flexibility of molecular chains, FFV and CED can significantly affect the *T*_g_ and theoretical modulus. High molecular chain flexibility, a high FFV and a small CED will result in a low *T*_g_ and modulus.

(3) The simulation results of *T*_g_ and Young’s modulus of the DGEBA/MTHPA and DGEBA/NA systems were consistent with the experimental results, which verifies the accuracy of the crosslinking model of the anhydride-epoxy system in this paper. This can provide a scientific basis for the optimization of anhydride-epoxy systems in the field of high voltage insulation.

## Figures and Tables

**Table 1:** Initial parameters of DGEBA/MTHPA and DGEBA/NA epoxy resin models.

Structure Models	Molecular Number of			Amorphous Models		Crosslinked Models		Volumetric Shrinkage
				Volume	Density	Volume	Density	
	DGEBA	MTHPA	DGEBA-MTHPA/NA	Å^3^	g/cm^3^	Å^3^	g/cm^3^	%
DGEBA/MTHPA	8	18	2	9782.613	1.143	9693.979	1.153	0.91%
DGEBA/NA	8	18	2	9387.993	1.184	9224.818	1.205	1.74%

**Table 2:** The cohesive energy density (CED) of the epoxy system.

Epoxy System	DGEBA/MTHPA	DGEBA/NA
CED (J·cm^−3^)	464.12 ± 21.43	475.50 ± 24.33

**Table 3:** Free volume of the epoxy system.

Epoxy System	*V*_f_ (Å^3^)	*V*_total_ (Å^3^)	FFV (%)
DGEBA/MTHPA	1616.96 ± 67.88	9697.1 ± 66.52	16.67 ± 0.59
DGEBA/NA	1285.69 ± 42.66	9067.72 ± 35.84	14.18 ± 0.43

**Table 4:** Simulation and experimental results of the *T*_g_ calculated by the specific volume-temperature method (SV) and the MSD-temperature method (M).

Method	DGEBA-MTHPA		DGEBA-NA	
	*T*_g_ (K)		*T*_g_ (K)	
Simulation	SV	399.03	SV	421.02
	M	398.26	M	419.46
Experiment		401.62		427.48

**Table 5:** The *T*_g_ of the epoxy systems.

Epoxy System	Young’s Modulus (GPa)	Bulk Modulus (GPa)	Shear Modulus (GPa)	Poisson’s Ratio
DGEBA/MTHPA	4.14 ± 0.18	3.5 ± 0.86	1.6 ± 0.06	0.29 ± 0.06
DGEBA/NA	4.87 ± 0.41	3.89 ± 0.47	1.7 ± 0.46	0.3 ± 0.03
